# Development of a Cost-Efficient and Glaucoma-Specialized OD/OC Segmentation Model for Varying Clinical Scenarios

**DOI:** 10.3390/s24227255

**Published:** 2024-11-13

**Authors:** Kai Liu, Jicong Zhang

**Affiliations:** 1 School of Biological Science and Medical Engineering, Beihang University, Beijing 100083, China; liukai2918@buaa.edu.cn; 2 Beijing Advanced Innovation Centre for Biomedical Engineering, Beihang University, Beijing 100083, China; 3 Department of Computer Science, City University of Hong Kong, Hong Kong 98121, China; 4 Hefei Innovation Research Institute, Beihang University, Hefei 230012, China

**Keywords:** color fundus image, glaucoma-specialized model, progression tracking, OD/OC segmentation, adapted multi-scenarios

## Abstract

Most existing optic disc (OD) and cup (OC) segmentation models are biased to the dominant size and easy class (normal class), resulting in suboptimal performances on glaucoma-confirmed samples. Thus, these models are not optimal choices for assisting in tracking glaucoma progression and prognosis. Moreover, fully supervised models employing annotated glaucoma samples can achieve superior performances, although restricted by the high cost of collecting and annotating the glaucoma samples. Therefore, in this paper, we are dedicated to developing a glaucoma-specialized model by exploiting low-cost annotated normal fundus images, simultaneously adapting various common scenarios in clinical practice. We employ a contrastive learning and domain adaptation-based model by exploiting shared knowledge from normal samples. To capture glaucoma-related features, we utilize a Gram matrix to encode style information and the domain adaptation strategy to encode domain information, followed by narrowing the style and domain gaps between normal and glaucoma samples by contrastive and adversarial learning, respectively. To validate the efficacy of our proposed model, we conducted experiments utilizing two public datasets to mimic various common scenarios. The results demonstrate the superior performance of our proposed model across multi-scenarios, showcasing its proficiency in both the segmentation- and glaucoma-related metrics. In summary, our study illustrates a concerted effort to target confirmed glaucoma samples, mitigating the inherent bias issue in most existing models. Moreover, we propose an annotation-efficient strategy that exploits low-cost, normal-labeled fundus samples, mitigating the economic- and labor-related burdens by employing a fully supervised strategy. Simultaneously, our approach demonstrates its adaptability across various scenarios, highlighting its potential utility in both assisting in the monitoring of glaucoma progression and assessing glaucoma prognosis.

## 1. Introduction

Glaucoma is a prominent contributor to blindness, as it causes pathological damage to retinal cells that results in visual impairment or even permanent blindness [1]. Regular glaucoma screening facilitates timely therapeutic interventions, while precise monitoring of the progression and assessment of the prognosis of glaucoma are also crucial for mitigating further vision loss [2]. Nonetheless, a significant obstacle arises in acquiring progression-related features, such as the cup-to-disc ratio, as this process heavily relies on the segmentation results of glaucoma-confirmed fundus images. Regrettably, few models have been dedicated to addressing this task.

Currently, multiple imaging methods are available to obtain markers of glaucoma progression and prognosis. Optical coherence tomography offers deep and detailed insight into retinal layers by a non-invasive method. Therefore, to assist in evaluating glaucoma progression, in our previous study [3], we leveraged OCT image to obtain OCT image-based biomarkers by accurate segmentation of retinal layers. However, OCT imaging is primarily only accessible in developed regions because of the high cost [4]. Moreover, evaluating the development of glaucoma necessitates the consideration of various clinical indicators, such as visual field measurements, thickness of the retinal nerve fiber layer, and optic cup-to-disc diameter ratio, among others. However, retina-layer-based biomarkers derived from OCT images contribute only to parts of the related biomarkers. Meanwhile, color fundus imaging has proven its ability in monitoring fundus conditions, offering a cost-effective advantage [5]. Although the segmentation of OD and OC can be used to derive biomarkers, such as the cup-to-disc diameter ratio, which plays a key role in assessing glaucoma progression, manual delineations of the contours of the optic disc (OD) and optic cup (OC), however, continue to be time-intensive and rely on clinical expertise, particularly for glaucoma samples [6]. Therefore, the development of an automatic segmentation tool holds importance in aiding the medical community, especially in regions with a shortage of ophthalmologists.

**Figure 1:**
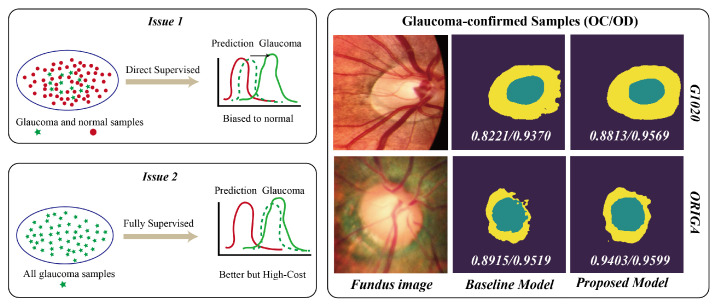
Illustrations of our motivation and the promising performance of our proposed model.

As illustrated in Figure 1, it is evident that most fundus image segmentation models, employing a directly supervised strategy, are not optimal for assisting in tracking glaucoma progression and prognosis assessment. These models are primarily tailored to screening or detection scenarios characterized by a large proportion of normal fundus samples, with a relatively minor portion of glaucoma fundus samples [7]. Therefore, the limited capacity of the models yields suboptimal performances on glaucoma samples. In the scenario of assessing glaucoma progression, the models are presented with the samples that have been confirmed to exhibit glaucoma. Therefore, if models exhibit poor performances on glaucoma samples, they are not the optimal choice for assisting in the assessment of glaucoma progression. Additionally, establishing these models requires both glaucoma and normal samples with pixel-level annotations. Moreover, as widely acknowledged, fully supervised models exhibit remarkable performances when benefiting from sufficient annotations, but they are constrained by the high costs associated with collecting and annotating glaucoma samples. Therefore, developing a cost-efficient model with low-cost annotations is appealing.

Currently, there is sufficient availability of normal fundus images with pixel-level annotations that are accessible at low cost. Notably, compared with other retinal diseases (e.g., age-related macular degeneration), glaucoma samples do not exhibit novel or distinct lesions that might disrupt layouts and patterns. The differences between the normal and glaucoma classes primarily reside in the sizes and morphologies of the OD and OC. This key characteristic ensures the foundational performance of transfer learning [8]. Therefore, it is beneficial to exploit shared knowledge from normal samples based on the similarities between the two classes. However, if the proposed model struggles to disentangle the relationship, the model will inevitably capture both shared and unshared features simultaneously, resulting in poor performance. Therefore, adapting inherent similarities and dissimilarities between the two classes can potentially avoid this limitation. We argue that the relationship between the two classes can be established by the utilization of low-cost annotations at both the style-level and domain-level.

The models, assisting in glaucoma progression tracking and prognosis assessment, should yield optimal performances on glaucoma-confirmed samples, while the prediction distributions of most existing models employing a directly supervised strategy with mixed glaucoma and normal fundus images tend to bias a normal distribution. Additionally, establishing a fully supervised model is restricted by the high cost of collecting and annotating glaucoma fundus images. To alleviate the issue, we exploit shared knowledge from low-cost pixel-level annotated normal fundus images, capturing general and glaucoma-related features by the acquisition of insights into the relationship between the two classes, accomplished by the utilization of low-cost style-level and domain-level annotations. The right-hand illustrations exhibit two test examples from two datasets. The baseline model was directly supervised by normal samples without the proposed modules. Moreover, in clinical practice, it is common to encounter various scenarios. Specifically, we may only have access to a limited number of annotated glaucoma samples, alongside an extensive collection of unannotated glaucoma samples. Effectively leveraging these resources is critically important in clinical practice. Therefore, developing a model that can effectively adapt to diverse clinical scenarios is equally important. Note that the term “pixel-level annotations” refers to annotations of the individual pixels within each image, including the background, optic disc, and optic cup. “Domain-level annotations” denote the classification of each image into a specific domain, such as the glaucoma or normal domain. In contrast, “style-level annotations” describe the stylistic characteristics of each image, distinguishing between glaucoma-style and normal-style presentations.

To summarize, the motivations for this study include the following three aspects: (1) existing OD/OC segmentation models are not optimal for assisting in the assessment of glaucoma progression; (2) developing a fully supervised, glaucoma-specific OD/OC segmentation model from scratch has a high cost; (3) achieving adaption to various scenarios encountered in clinical practice. We hereby clarify the similarities and differences between this study and our previous study [3]. Both studies are related to assisting in the assessment of glaucoma progression. Our previous study primarily addressed retinal layer segmentation on of OCT images, which incurs high acquisition costs. In contrast, this study concentrated on color fundus images, obtaining OD- and OC-based glaucoma-related biomarkers, a low-cost imaging method that is particularly suitable for resource-limited regions. Moreover, regarding the framework, while both studies employ adversarial learning, this study introduces an additional component, style-based contrast learning, which focuses on the combination of style-based contrast learning and domain adversarial learning, rather than exploiting multi-level features from various encoding and output spaces, which was extensively explored in the previous study. Therefore, with the collaboration between contrast and adversarial learning, the model presented in this study adapts to three clinical scenarios, enhancing its clinical significance. In contrast, the model proposed in our previous study [3] was limited to a single scenario.

Therefore, we propose a novel annotation-efficient model by exploiting low-cost annotations, including pixel-level annotations for normal samples, as well as style-level and domain-level annotations for normal and glaucoma samples. The proposed model can capture general features from low-cost annotated normal fundus images, simultaneously capturing glaucoma-related features from style-level and domain-level annotations. We employ a Gram matrix to encode the style information of the feature maps to capture style gaps. In addition, domain-level supervised modules in the feature space and output space are employed to bridge domain-level gaps between the two classes. The architecture of our proposed model consists of the following two components: The first part, shared by normal and glaucoma images, is dedicated to being supervised with pixel-level annotated normal images (and annotated glaucoma images, if available). The role of the second part involves evaluators, explicitly assessing style and domain gaps between the normal and glaucoma classes dedicated to being supervised with style-level and domain-level annotations. Therefore, the annotation-efficient strategy encourages the proposed model to generate results closely aligned with the glaucoma class by explicitly narrowing the domain and style gaps to the glaucoma class.

Therefore, in this study, the low-cost annotations come from two aspects: (1) the relative abundance of normal eye data in the population, making them more accessible than glaucoma samples; (2) compared to the significant alterations in the OD and OC in glaucoma cases since the introduction of lesions and the varying severities of glaucoma, the minimal changes in normal eyes facilitate the clear delineation of the OD and OC boundaries in the normal samples. Considering the few publicly available specialized datasets for this task, we adapted two public fundus-image-based datasets to simulate the various scenarios. We employed annotated normal images as the resource dataset, while glaucoma images were the target dataset used to validate our model. Our proposed model offers distinct advantages in three common scenarios: pixel-level annotated normal or glaucoma samples, and both annotated normal and glaucoma samples with pixel-level annotations.

The contributions of this work are summarized as follows:

1. We demonstrate a concerted effort to target glaucoma-confirmed samples by proposing a glaucoma-specific model, mitigating the inherent issue of bias in most existing direct supervised models with mixed annotated glaucoma and normal fundus samples, yielding an optimal performance in OD/OC segmentation in fundus images, assisting in glaucoma progression tracking and prognosis assessment.
2. Our proposed model exhibits a cost-efficient performance by exploiting annotated normal images, mitigating the high cost of employing fully supervised pixel-level annotated glaucoma samples. Moreover, our proposed model explicitly establishes the relationship between the two classes by style contrastive and domain adversarial learning, improving predictions for glaucoma by narrowing the style-level and domain-level gaps, preventing deterioration in performance due to unshared features.
3. The disentangled relationship established by our proposed model facilitates its adaptability in three common scenarios, wherein normal or glaucoma samples, or both are provided with pixel-level annotations. Notably, our proposed model demonstrates cost-effectiveness by yielding an enhancement in the overall performance simply by increasing the number of normal samples.
4. We conducted experiments employing two public datasets to mimic various common scenarios. The results demonstrate that our proposed model yields a superior performance over the baseline model, approaching the model with supervision by the annotated glaucoma samples. Moreover, the proposed model accurately delineates the contours of the OD and OC, facilitating the derivation of glaucoma progression-related features.

## 2. Related Work

In this section, we briefly summarize OD and OC segmentation approaches and cost-efficient strategies for medical images.

### 2.1. OD and OC Segmentation Approaches for Fundus Images

Numerous classical approaches have been proposed for OD and OC segmentation in fundus images. These approaches attempt to utilize discriminate features to classify the OD and OC. Several methods have been explored, including edge or morphology detection [9,10,11], level set [12,13,14], template matching [15,16,17], and graph cut [18], as well as super-pixel classification [19,20,21]. The advantage of these approaches lies in their ability to detect well-interpretable features, which are critical for clinical applications. However, these hand-crafted features rely heavily on prior expert knowledge [7]. In addition, the generalization ability of classical methods is limited, as they struggle to adapt to variations in appearance, anatomy, and domain shifts.

Recently, deep learning-based methods have shown considerable potential in medical image tasks, and several notable frameworks have been proposed for the task of segmenting ODs and OCs [22]. Notably, convolutional neural networks have been extensively employed, owing to their capability in automatically capturing discriminator features from raw fundus images [23,24]. Subsequently, U-Net [25] has been adapted for OD and OC segmentation tasks, employing an encoder–decoder architecture to effectively capture contextual information and preserve spatial details. Moreover, the incorporation of advanced modules, such as residual blocks, dense connections, and dilated convolution, has resulted in notable enhancements in segmentation accuracy [26,27]. Additionally, U-Net variations, including M-Net [28], U-Net++ [29], and CE-Net [30], yield further improvements in performance. Attention-based models with spatial- and channel-wise attentions have demonstrated efficacy in distinguishing OD and OC boundaries [31,32].

However, most developed models are not optimal for assisting in the tracking of progression and the assessment of prognosis for glaucoma. These models primarily focus on early detection, establishing models with mixed samples that mimic common screening scenarios by combining a larger number of normal samples with a smaller number of glaucoma samples, such as the ESPERANZA dataset (113 glaucoma vs. 1333 normal) [33] and the RIM-ONE-R1 dataset (40 glaucoma vs. 118 normal) [34]. The biased datasets result in suboptimal performances on glaucoma-confirmed samples.

### 2.2. Cost-Efficient Strategies in Medical Image

To mitigate the resource-intensive burden of annotating and collecting samples, especially for medical image analysis tasks, various cost-efficient strategies have been proposed [35,36] demonstrating promising economic efficacy [37,38,39]. The pretrained model, a common cost-efficient method, trains a model with low-cost datasets, followed by fine-tuning the initialized, optimized model on a limited target dataset [40]. Christopher et al. proposed a high-performance model with a rapid training speed, employing a pretrained model strategy to detect glaucomatous optic neuropathy using a public nature-based image dataset, thus mitigating expenses by annotating fundus images [41]. However, capturing shared features from nature images is not the optimal choice for medical image tasks because of the significant disparities between nature- and medical-based images. Gomez et al. proposed a cost-efficient model that exploits valuable resources from a separate medical image dataset for the targeted task of OD segmentation in fundus images [33]. Therefore, pretrained models can serve as an annotation-efficient method by exploiting resources in low-cost datasets.

However, a limitation of pretrained models is that the source dataset does not participate in the training stage of the downstream task, resulting in the model capturing shared and noisy features simultaneously [42,43]. Therefore, Zhang et al. proposed a source-free model for diabetic retinopathy detection by incorporating source samples during the training stage, encouraging the model to generate target-style features from unannotated datasets [44]. Commonly, large domain gaps between the source and target datasets deteriorate performance; thus, domain adaptation models are introduced in medical image tasks to narrow the domain gaps between the source dataset and the target dataset [45,46]. Lei et al. proposed an unsupervised domain adaption model to effectively narrow domain gaps, encouraging the model to capture the target-domain knowledge from the source dataset, yielding a superior performance in the segmentation of ODs and OCs [47]. Moreover, Keaton et al. combined domain adaptation with few-shot learning to segment cellular instances by a new contrastive loss, exhibiting a promising performance on a minimal number of new annotated samples [48]. Similarly, Feng et al. proposed a domain adaptation module with consistency match, exploiting knowledge from low-cost relevant datasets to the task of pneumonia diagnosis [49].

With the benefits of sufficient annotated datasets, the fully supervised method has demonstrated the achievement of promising performances, but its extension to a glaucoma-specialized model is restricted by the limited availability of annotated glaucoma samples. Therefore, it is beneficial to exploit resources from low-cost annotations.

## 3. Methods

In this section, we first provide an overview of our proposed model, followed by details on the style and domain collaborative supervision learning scheme. Finally, we provide in-depth insight into the overall training procedure.

### 3.1. Overview of the Proposed Model

**Figure 2:**
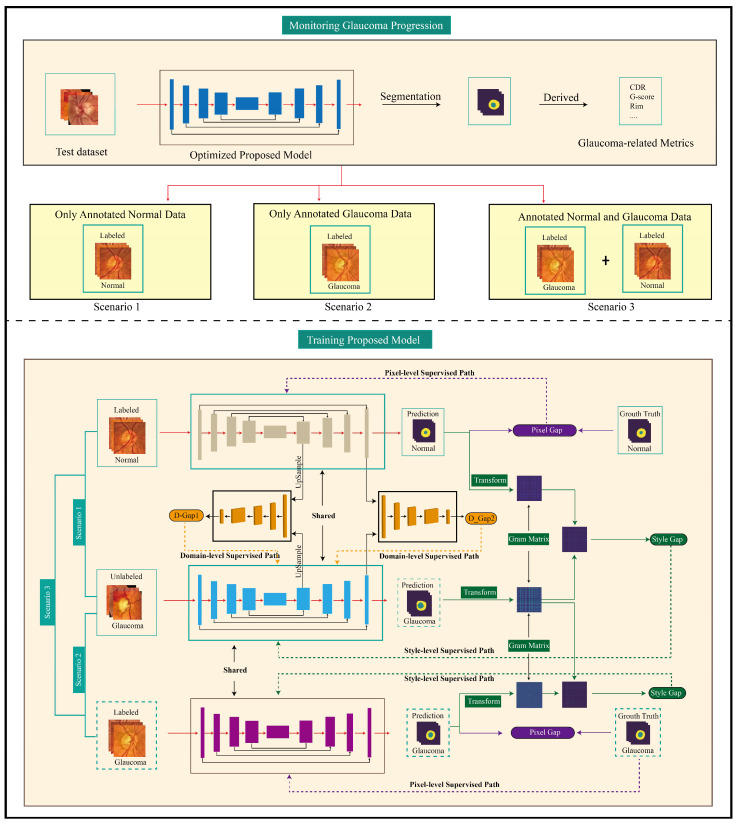
The proposed model adapts three common situations (Scenario 1: only normal images have pixel-level annotations; Scenario 2: only glaucoma images have pixel-level annotations; Scenario 3: both normal and glaucoma images have pixel-level annotations). The pixel-level annotated normal fundus images (and pixel-level annotated glaucoma fundus images, if available) are utilized to capture general features with the pixel-level supervised annotations. The glaucoma fundus images (without pixel-level annotations) are utilized to capture glaucoma-related features with the style-level and domain-level supervised annotations. The proposed model encompasses a pixel-level supervised path that aims to generate pixel-level prediction results by soft dice loss; the style-level supervised path is designed to encourage the generation of pixel-level prediction results similar to glaucoma-style features by narrowing style gaps; and the domain-level supervised path encourages the generation of pixel-level prediction results close to the glaucoma-domain at various domain levels by narrowing domain gaps. For detailed frameworks corresponding to each scenario, please refer to three separate images (Figures S2–S4) in the Supplementary Materials.

Our proposed model, as illustrated in Figure 2, incorporates shared feature extraction with three distinct output pathways. One pathway is dedicated to capturing pixel-level features, while the remaining two pathways are designed to capture class-level features, including style and domain features. The optimization process is as follows: The shared model produces encoding features and pixel-level predictions, as supervised by pixel-level annotations of normal or glaucoma images (or both, i.e., two-class images). Subsequently, a refinement stage collaboratively encourages the pixel-level predictions toward the glaucoma class by updating the shared model with style-level and domain-level supervised annotations, narrowing the style and domain gaps between the two classes.

### 3.2. Style and Domain Collaborative Supervision Learning

We began by establishing the initialized, optimized segmentation model (S*) with pixel-level annotated images. The initialized model served as the base model to generate pixel-level prediction results for the next steps. We forwarded normal fundus images ($I_{n}$) (and annotated glaucoma images, if available) to the segmentation module and optimized it with the corresponding pixel-level labels ($Y_{n}$) by soft dice loss ($L_{seg}$), which is defined as follows:

(1) $$L_{seg}\left(I_{n},Y_{n}\right)=1-\frac{2*\left(S\left(I_{n}\right)*Y_{n}\right)}{S{\left(I_{n}\right)}^{2}+{Y_{n}}^{2}}.$$

Subsequently, we forwarded $I_{g}$ and $I_{n}$ images to the initialized, optimized module, obtaining pixel-level prediction results for the normal images, $S\left(I_{n}\right)$, and glaucoma images, $S\left(I_{g}\right)$. These results serve as inputs for the latter. The architecture of the segmentation modules is described in detail in Section 3.5, on our proposed framework. In the following steps, we focus on shifting the distribution of the results toward the glaucoma class by utilizing style and domain annotations.

### 3.3. Style Contrastive Learning

Inspired by prior knowledge, the difference between the color fundus images of normal and glaucoma eyes can be regarded as imaging style variations, as glaucoma mainly causes morphological alterations like optic cup collapse. However, the challenge lies in quantifying these stylistic differences between the two images. In this study, we adopted a contrast learning-based strategy. Unlike adversarial learning, which relies on a style discriminator, contrast learning directly assesses style gaps with a similarity assessment function. Therefore, this strategy is easy to implement in practice. Moreover, it is suitable when only one class is present, especially in Scenario 2, with only glaucoma samples, for which the training of adversarial learning discriminators becomes unfeasible, as it requires normal and glaucoma samples.

To effectively capture the stylistic features of an image, we drew inspiration from the style loss of style transfer learning, for which the stylistic information of an image is encoded through a style matrix, notably a Gram matrix. This matrix can be interpreted as covariance representation for features within an image. This is achieved by computing the inner product between a given feature and the remaining features within an image. This matrix captures the correlations among different features, specifically their co-occurrence patterns. Since images of varying styles exhibit different feature co-occurrences, the matrix effectively delineates the stylistic attributes associated with each image.

The style supervision module, denoted as G, serves as a style gap evaluator responsible for narrowing the style gap by supervising style-level annotations. The channel-wise Gram matrix is employed to encode the style information of feature maps. We first obtained Gram matrices of the prediction results, $S\left(I_{n}\right)$ and $S\left(I_{g}\right),$ as per the following steps: initially, we transformed a raw feature map with dimensions of H × W × C (height × width × channel) reshaped into a new feature map with dimensions of HW × C. Subsequently, we transpose the reshaped feature map (C × HW) and perform matrix multiplication with the reshaped feature map (HW × C) to obtain a channel-wise Gram matrix (C × C). The Gram matrix is defined as follows:

(2) $$Gram\left(I_{x}\right)=R{\left(S\left(I_{x}\right)\right)}^{T}\times R\left(S\left(I_{x}\right)\right),\;$$

where the Gram matrix of feature maps $I_{x}$ is denoted as $Gram\left(I_{x}\right)$; $R\left(S\left(I_{x}\right)\right)$ represents the reshaped $S\left(I_{x}\right)$; and *x* is the normal or glaucoma images. The difference between the Gram matrices of the normal and glaucoma prediction results across all positions can be treated as the style gap loss ($L_{style}$) between the two classes. The style loss is defined as follows:

(3) $$L_{style}=\underset{\left\{I_{g}\in S_{g},I_{n}\in S_{n}\right\}}{\sum }\frac{\sum_{\left\{r\in C,c\in C\right\}}{\left(Gram{\left(I_{n}\right)}^{r,c}-Gram{\left(I_{g}\right)}^{r,c}\right)}^{2}}{C*C},$$

where *r* and *c* denote the row and column of the Gram matrix. The detailed style supervision steps are as follows: Firstly, the results derived from the initialized, optimized segmentation module, S*, are conveyed to the corresponding channel-wise Gram matrix according to Equation (3). Subsequently, the mean squared error between the Gram matrices of the normal prediction result and the target style glaucoma prediction result is assessed according to Equation (4), followed by normalization with the total number of elements in the style map. Next, the obtained style loss is propagated backward to the segmentation module, encouraging S* to produce results that approximate the glaucoma style.

### 3.4. Domain Adversarial Learning

Only narrowing the style gap is insufficient for dense pixel prediction tasks [3]. Therefore, we further establish the relationship with domain-level annotations. Employing a fully supervised learning strategy and training an OD/OC segmentation model using only pixel-level labeled normal color fundus images, the generated results are inevitably biased toward the normal domain and away from the glaucoma domain. This bias leads to poor performance in our target task: OD/OC segmentation in glaucoma-confirmed samples. Therefore, if the generated results align closely with the glaucoma domain, it is more likely to achieve improved segmentation results on glaucoma-confirmed samples. Inspired by previous work indicating that generator adversarial learning can effectively narrow the generated distribution to align with the target distribution [50], we introduced domain adversarial learning to refine the generated results, encouraging them to more closely match the glaucoma distribution.

Commonly, generator adversarial learning consists of a generative part and a discriminator part. In this study, we used the segmentation module (denoted as $S\left(•\right)$) as a generator, generating segmentation results that closely approximated the target glaucoma domain. The discriminator, in its original role, evaluates the gap between the generated fake results and the real results, thus guiding the generator to produce more realistic results. In this study, we employed a domain discriminator to assess the domain gap between the generated segmentation results and the target glaucoma domain to promote the generation of results by the segmentation module to be as close as possible to the glaucoma domain. The domain discriminator module, denoted as *D*(•), serves as a domain assessor responsible for reducing domain gaps between the two classes, which is established using a fully convolutional neural network consisting of four convolution layers (refer to Table 1 for details on the framework). The primary objective of the modules is to classify the input domains using the domain discriminator loss ($L_{D}$), as in Equation (4), and binary cross-entropy loss, which can be written as follows:

(4) $$L_{D}=-\underset{\left\{i\in e,o\right\}}{\sum }\underset{\left\{I_{g}\in T_{g},I_{n}\in T_{n}\right\}}{\sum }\left(1-Z\right)\log\left(D{\left(S\left(I_{n}\right)\right)}_{i}\right)+Z\log\left(D{\left(S\left(I_{g}\right)\right)}_{i}\right),$$

where $T_{g}$ and $T_{n}$ correspond to the sample sets of glaucoma and normal fundus images, respectively; $D_{i}$ is the domain discriminator module in the encoding space or output space; and *Z* is assigned as 0 or 1 depending on the domain label of the segmentation soft-max map.

High-dimensional encoding spaces contain visual features of a global size, while the low-dimensional output space contains features that are directly related to the segmentation features, including layout and textures. Although due to constraints related to computational resources, we solely employed two domain discriminators to capture two-level domain gap losses, one from the encoding feature space (Up1 encoding space, as shown in Table 1, with dimensions of 256×32×32. Prior to being input into the domain discriminator, it is upsampled to 256×256×256) and another from the output space (dimensions of the output feature are 3×256×256), for capturing different glaucoma-domain features from two different aspects.

Based on the initialized optimized segmentation module supervised by pixel-level annotated normal samples, we obtain the prediction results and encoding features of the normal images, $S\left(I_{n}\right)$, and glaucoma images, $S\left(I_{g}\right)$, by inputting the glaucoma samples, $I_{g}$, and normal samples, $I_{n}$. Then, we forward the prediction results and encoding features from the segmentation module to the corresponding domain modules to obtain the domain discriminator losses according to Equation (4) and to optimize the corresponding domain modules to improve the ability of the discriminator.

To evaluate the domain gap loss from the normal domain to the glaucoma domain, we treated the glaucoma domain as the target domain-level label for the normal images. To obtain the two-level domain gap loss ($L_{domain}^{i}$), we forwarded the segmentation results, ($S{\left(I_{n}\right)}_{o}$), and encoding feature maps, $\left(S{\left(I_{n}\right)}_{e}\right)$, of the normal images, ($I_{n}$), generated by the segmentation module, ($S\left(•\right)$), to the corresponding domain discriminator module, ($D{\left(•\right)}_{i})$. The domain gap loss is defined as follows:

(5) $$L_{domain}^{i}=\sum_{h,w}\log\left(D{\left(S{\left(I_{n}\right)}_{i}\right)}_{i}\right),i\in \left(e,o\right),$$

(6) $$L_{domain}=-\underset{I_{n}\in S_{n}}{\sum }[w_{e}*L_{domain}^{e}+w_{o}*L_{domain}^{o}],$$

where $L_{domain}^{i}$ denotes the domain gaps from the *i* space; $L_{domain}^{e}$ and $L_{domain}^{o}$ denote the domain gaps from the encoding space and output space, respectively; $w_{e}$ and $w_{o}$ are the corresponding weight coefficients to balance the impact of the two losses. In this study, $w_{e}$ and $w_{o}$ were 0.2 and 0.8, respectively.

In detail, the steps of the domain supervision learning are as follows: We first obtained the domain gap losses according to Equation (6). Subsequently, we updated the initialized optimized segmentation module using the domain gap losses. As gradients stemming from the *D* module are back-propagated to the segmentation module, transferring the segmentation results close to the glaucoma class at the domain level, and encouraging S* to capture glaucoma-related features. Moreover, we should progress in improving the capacity of the domain supervision modules to match the capacity of the segmentation module in training.

### 3.5. Objective Function for Our Proposed Model

The goal of our proposed model was to generate pixel-level results as close as possible to the glaucoma class while simultaneously capturing as many general features as possible. Therefore, we formulated a task with an objective function with three distinct losses: a segmentation dice loss for capturing the pixel-level features, and style and domain gap losses for capturing the style- and domain-level features simultaneously. Therefore, the overall object function can be written as follows:

(7) $$L\left(I_{N},I_{G}\right)=w_{seg}L_{seg}\left(I_{N}\right)+w_{style}L_{style}\left(I_{G},I_{N}\right)+w_{domain}L_{domain}\left(I_{G},I_{N}\right),$$

where $L_{seg}$ denotes the segmentation dice loss supervised by the pixel-level ground truth of the normal images (and annotated glaucoma images, if available); $L_{style}$ and $L_{domain}$ are the style and domain gap losses between the normal and glaucoma classes, respectively; $w_{seg}$, $w_{style}$, and $w_{domain}$ are the corresponding weights to adjust the impacts of the different losses; and $I_{N}$ and $I_{G}$ denote fundus images from the normal and glaucoma classes, respectively. During the first learning stage, we set the weight of the segmentation and style contrastive learning losses to 1.0 and 0.05, respectively, while the weight of the adversarial learning loss was fixed at 0.0. In the second learning phase, the weight of adversarial learning loss was increased to 1.0, while the remaining two weights were both set to 0.0.

### 3.6. Overtraining Procedure

To achieve the objective of our proposed model, we aimed to minimize the segmentation loss and style loss for the source normal images in the segmentation module, while concurrently maximizing the probability of predictions being classified as target glaucoma domains. Inspired by generative adversarial networks [50,51], the min–max training strategy was employed to facilitate the optimization of our proposed model, which is defined as follows:

(8) $$\underset{D}{max}\underset{Seg+Sty}{\;min}\;L\left(I_{g},I_{n}\right).$$

To encourage the proposed model to simultaneously capture general and glaucoma-related features, we minimized the segmentation error and style gaps using the soft dice loss and style gap loss within the segmentation module (*Seg*) and the style supervision module (*Sty*), maximizing the probability of the segmentation results being considered as in the glaucoma domain within the domain supervision modules (*D*).

The detailed training procedure, as depicted in the following flowchart for Algorithm 1, mainly includes a dual-step iterative process. Step one: We fix the parameters of the domain modules and update the parameters of the segmentation module. The normal images, $I_{n}$, are forwarded to the segmentation module, optimizing it by supervision with pixel-level annotations, $Y_{i}$, according to the soft dice loss. We further update the segmentation module by backpropagating gradients from the style and domain transfer modules with the style and domain gaps. Step two: We fix the parameters of the segmentation module while updating the parameters of the domain modules by supervising with domain-level annotations to improve the capability of the domain modules. The iteration process between the segmentation module and transfer modules will not stop until achieving relatively low and stable gap losses in the style and domain.
**Algorithm 1**: Training Conducted by Our Proposed Model**Input**: A batch of ($I^{n}$, $Y^{n}$) from the annotated normal dataset, $D_{\mathrm{AN}}$ (as well as ($I^{g}$, $Y^{g}$) from the annotated glaucoma dataset, $D_{\mathrm{AG}}$, if available) and $I^{g}$ from the unannotated glaucoma dataset $D_{\mathrm{UG}}$. **Output**: Trained segmentation network, $N_{S}$, and domain supervision network, $N_{D}$, with parameters $\theta_{S}$ and $\theta_{D}$, respectively.1: **While** not converge **do**2:    ($I^{n}$, $Y^{n}$), $I^{g}$ ← sampled from $D_{\mathrm{AN}}$ and $D_{\mathrm{UG}}$3:    **Step 1**: Optimize the segmentation network $N_{S}$, fixed $N_{D}$4:        Generate encoding features $F{\left(I^{n}\right)}_{e}$ and $F{\left(I^{g}\right)}_{e}$ and segmentation results $S{\left(I^{n}\right)}_{o}$
         and $S{\left(I^{g}\right)}_{o}$ from $N_{S}$5:        Generate output results of domain supervision module: D($F{\left(I^{n}\right)}_{e}$) and D($S{\left(I^{n}\right)}_{o}$)6:        Calculate segmentation loss $L_{seg}$, as in Equation (1)7:        Calculate style loss $L_{style}$, as in Equation (3)8:        Calculate domain gap loss $L_{domain}$, as in Equation (6) 9:        Update $\theta_{S}$ ← – ($w_{seg}*L_{seg}$ – ($w_{style}*L_{style}$ + $w_{domain}*L_{domain}$))10:     **Step 2**: Optimize the domain supervision network $N_{D}$, fixed $N_{S}$11:         Generate the encoding features and segmentation results for the normal samples ($F{\left(I^{n}\right)}_{e}^{*}$
$\mathrm{and}\;S{\left(I^{n}\right)}_{o}^{*}$ and glaucoma samples ($F{\left(I^{g}\right)}_{e}^{*}$ and $S{\left(I^{g}\right)}_{o}^{*}$) from Step 1’s optimized $N_{s}^{*}$12:         Calculate discriminator loss $L_{D}$, as in Equation (4)13:         Update $\theta_{D}$ ← – ($L_{D}$)14: **end while**15: **return** Trained network $N_{s}^{**}$ and $N_{d}^{**}$Note: During Scenario 2, without annotated normal samples, the proposed model is reduced to a single-style contrastive learning model, only executing Step 1 and setting $w_{domain}$ to 0.

## 4. Experiments

### 4.1. Dataset

#### 4.1.1. Overview of Dataset

We adopted two glaucoma detection datasets to mimic various situations to validate our proposed model, including two fundus image datasets. The first dataset (ORIGA [52]), widely used in related research, contains 650 images (482 normal and 168 glaucoma) and was released by the Singapore Eye Research Institute. All images have pixel-level annotations for the OD and OC, annotated by an ophthalmologist. The second dataset (G1020 [53]), similar to real clinical conditions without the specific imaging constraints, contains 1020 images (296 glaucoma and 724 normal) and was released by Technische Universitat Kaiserslautern German Research Center for Artificial Intelligence GmbH. While only 790 images have both OD and OC pixel-level annotations, performed by a single ophthalmologist. In this study, we exclusively utilized samples that included pixel-level annotations of the optic disc and optic cup as glaucoma-related biomarkers, specifically CDR and G-score, which require two annotations for an accurate implementation. We utilized the normal images as the source dataset to exploit resources and glaucoma fundus images as the target dataset to validate our proposed model. A summary of the datasets is provided in Table 2.

#### 4.1.2. Preprocessing of Images

All preprocessing steps used for our images are shown in Figure S5, including two distinct preprocessing steps. To focus on the region of interest regions (ROIs), we performed direct cropping of the OD and OC regions in the fundus images, excluding the remaining parts. We utilized the location labels of the OD region and extracted a region 200 pixels away from the boundary of the OD region. Furthermore, the contrast degrees of the raw images exhibit variation and tend to be relatively low for some images, posing a challenge in determining the boundaries of ODs and OCs. We employed the adaptive histogram equalization algorithm [54], achieving a suitable performance that effectively mitigated indistinct contours of ODs and OCs.

### 4.2. Experimental Configuration

To ensure a fair comparison of all models and all three scenarios, we split the dataset based on the following method: For the adapted ORIGA dataset, we utilized 482 normal samples as the training source dataset and 100 glaucoma samples as the training target dataset. Additionally, the remaining 68 glaucoma samples were reserved as the validation dataset. For the adapted G1020 dataset, 554 normal and 136 glaucoma samples were utilized as the training source dataset and target dataset, respectively, while 100 glaucoma samples were allocated as the validation dataset.

A detailed framework is provided in Section 3.5 on the proposed network. The proposed model’s input image size is 256 × 256 × 3 (height × width × channel) with a batch size of 2. The AdaBoost optimizer is employed to optimize the segmentation, initialized with a learning rate of 1 × 10^−4^, momentum of 0.9, and betas of 0.9 and 0.99. StepLR with a gamma of 0.95 is employed to decay the learning rate. The soft-max results (256 × 256 × 3) from the segmentation serve as inputs of the style transfer module. The encoding feature maps (32 × 32 × 256) and outputs from the soft-max results (256 × 256 × 3) from the segmentation part serve as inputs of domain transfer modules. The AdaBoost optimizer with an initial learning rate of 1 × 10^−2^ and betas of 0.9 and 0.99 is utilized to optimize the domain transfer module.

### 4.3. Implementation Details and Evaluation Metrics

All experiments were implemented with PyTorch (pytorch-gpu version 1.8) [55] and trained using NVIDIA Tesla 4080 GPUs with 16 G memory (Gigabyte Graphics Card Company, Ningbo, China). The backbone framework of the segmentation was a customized U-Net, which is easily replaced by other advanced segmentation networks. Additionally, to effectively capture glaucoma-related features at both the style and domain levels, we incorporated weighted coefficients to balance the impact of the style and domain gap losses within the corresponding supervision paths.

In this study, we employed three evaluation metrics (Dice, cup-to-disc ratio (CDR), and G-score) based on the following considerations: the Dice coefficient, which reflects the original segmentation performance; cup-to-disc ratio (CDR), which serves as a biomarker associated with assisting in glaucoma development, derived from the original segmentation results; and G-score, which concurrently integrates the above two sides to provide a comprehensive assessment of the model’s performance.

To conduct a direct assessment of the segmentation performance of our proposed model, we employed the Dice coefficient, commonly used in medical image segmentation tasks. Moreover, it is more suitable for medical imaging than the intersection ratio commonly used in natural imaging, as it exhibits greater sensitivity to class imbalances (more sensitive to positive class). This sensitivity is particularly advantageous in medical imaging, where lesions (positive class) are often smaller than the background. It serves as a quantitative metric for assessing the similarity between prediction results and their corresponding ground truth by considering the metrics of true positive (*TP*), true negative (*TN*), and false negative (*FN*) in the segmentation results. A higher value of the Dice coefficient corresponds to a better segmentation performance. The formulation is defined as follows:

(9) $$Dice=\frac{2*TP}{TP+FP+TP+FN}.$$

To quantitatively evaluate the performance of our proposed model in monitoring glaucoma samples, we employed the cup-to-disc ratio metric, a common glaucoma-related feature that encompasses both vertical and horizontal directions. We computed the mean squared difference of the CDR between the prediction results and ground truth in two directions, which is defined in Equation (10), where the CDRs of the ground truth and prediction segmentation results are denoted as $CDR_{G}$ and $CDR_{P}$, respectively; and *n* represents the overall number of samples. A lower value of $CDR_{MSE}$ indicates a superior prediction performance.

(10) $$CDR_{MSE}\left(G,P\right)=\frac{\sum_{\left\{i\in g\right\}}{\left(CDR_{P}^{H}-CDR_{G}^{H}\right)}^{2}+\sum_{\left\{j\in g\right\}}{\left(CDR_{P}^{V}-CDR_{G}^{V}\right)}^{2}}{n}.$$

However, both the Dice and CDR assess the model’s performance from a singular perspective. To further evaluate the performance of our proposed model in assisting in monitoring glaucoma samples, we employed the G-score metric, as described in [52]. This metric offers a comprehensive evaluation by considering both the absolute difference in the CDR and the area of overlap between the prediction results and ground truth. A higher G-score value indicates an increase in the performance of the model, where $G_{score}$ denotes the G-score metric; $I_{pred}$ and $I_{gt}$ correspond to the prediction results and ground truth, respectively. The formulation is defined as follows:

(11) $$G_{score}=(((I_{pred}\cap I_{gt})/(I_{pred}\cup I_{gt}))\times 100)/\;2-\left(\left(\left|CDR_{pred}-CDR_{gt}\right|/CDR_{gt}\right)\times 100\right).$$

## 5. Experimental Results

### 5.1. Baseline Model Results

To comprehensively evaluate our proposed model, we established three pixel-level supervision baseline models (supervised by pixel-level annotations from normal, glaucoma, and both (i.e., two classes)) that corresponded to the three scenarios. The baseline models adopted the same framework as the segmentation part of our proposed model, but without the proposed modules. Additionally, the experimental settings were consistent, ensuring a fair comparison across all of the experiments.

#### 5.1.1. Pixel-Level Annotations from Normal Samples: Pixel-Supervised-N Baseline

To establish a base benchmark, we initially trained a model using only the pixel-level annotated normal fundus images (100). We considered this approach as the pixel-supervised-N baseline due to the exclusion of pixel-level annotated glaucoma images during the training stage. The experimental results, as shown in Table 3, demonstrate that the performance of the pixel-supervised-N baseline was not significantly poor, achieving a mean Dice (M-Dice) of 0.8879 for the OD, OC, and Rim segmentation results; mean square error of the CDR ($CDR_{MSE}$) of 0.0097; and G-score of 46.97. These modest results can be attributed to the inherent stylistic and domain similarities shared by normal and glaucoma fundus images. Nevertheless, the existing unshared and noised features prevent the pixel-supervised-N model from achieving an optimal performance.

#### 5.1.2. Pixel-Level Annotations from Glaucoma Samples: Pixel-Supervised-G Baseline

To establish a top-performance reference, we established a direct supervised model using the pixel-level annotated glaucoma fundus images (100), serving as the pixel-supervised-G baseline. The experimental results are shown in Table 3, which demonstrate that the pixel-supervised-G approach yielded a better performance than the pixel-supervised-N baseline across all three metrics, as follows: M-Dice (0.9107), $CDR_{MSE}$ (0.0028), and G-score (53.09). In addition, concerning the separated segmentation results for the OD, OC, and Rim, the pixel-supervised-G baseline also achieved a promising Dice score across all segmented regions. These promising results can be attributed to the same class of training dataset as the test dataset for the pixel-supervised-G method. However, the primary limitation of the pixel-supervised-G baseline lies in the high cost associated with annotating glaucoma images.

#### 5.1.3. Pixel-Level Annotations from Normal and Glaucoma Samples: Pixel-Supervised-G&N Baseline

To mimic datasets commonly utilized in glaucoma-screening or detection models, we established the pixel-supervised-G&N baseline by combining pixel-level annotated 100 glaucoma and 100 normal fundus images. The results are shown in Table 3. Leveraging pixel-level annotations from glaucoma samples, the mixed baseline showed a satisfactory performance (M-Dice: 0.9127), exhibiting a large improvement compared to the pixel-supervised-N baseline (M-Dice: 0.8879). However, it only achieved a small improvement compared to the pixel-supervised-G baseline (M-Dice: 0.9107). The direct reason is that the mixed pixel-supervised-G&N model displays a robust performance on both the normal and glaucoma samples simultaneously, decentralizing the model’s capacity. The more in-depth reason is that the straightforward supervised strategy fails to adequately capture the similarity and dissimilarity between the two classes. In summary, the high cost but marginal performance gains demonstrate that glaucoma-screening or detection models aren’t the optimal choice for assisting in monitoring the progression and assessing prognosis of glaucoma samples.

### 5.2. The Proposed Model’s Results

To explore the effect of our proposed model, we started with the single-level models with style- or domain-level annotations. Subsequently, we integrated the style and domain annotations to evaluate the effect of the collaborative supervision model. The experimental settings were kept the same as the baseline models.

#### 5.2.1. Single Style-Level Contrastive Learning Model

To demonstrate the superiority of our proposed model over the pixel-supervised-N baseline, we first conducted extensive experiments with the single-style level of supervision model with contrastive learning. The results, shown in Table 4, demonstrate that the style-level supervision model achieved superior performance. Specifically, the style-level supervision model achieved an M-Dice score of 0.8966, yielding an enhancement of 0.98% compared to the pixel-supervised-N baseline. The proposed model achieved both superior OC and Rim segmentation results. These promising results highlight the significant benefits of the style-level supervision module and the value of low-cost, style-level annotations, thereby narrowing segmentation error and style gaps with contrastive learning enables the model to capture general and glaucoma-style features simultaneously.

#### 5.2.2. Single-Domain-Level Adversarial Learning Model

In addition, we extensively experimented with the domain-level supervision model in the encoding space and output space by adversarial learning, since they contain different features. Furthermore, we used a patch size as the output dimension of the domain-level supervision module, since assessing the domain gaps at a suitable size prevents the model from losing important details and being affected by noise. All experimental settings were the same as the style of the supervision model. The experimental results, as shown in Table 4, prove the effect of the domain supervision model, exhibiting superior performance, with an M-Dice of 0.8975, and surpassing the pixel-supervised-N baseline. Furthermore, when only considering the encoding or output space domain information, the domain supervision learning model still outperformed the pixel-supervised-N baseline, with M-Dice values of 0.8923 and 0.8945, respectively. These results prove that exploiting knowledge from the domain information in the output and encoding spaces enables the model to capture domain-related features in different spaces.

#### 5.2.3. Collaborative Style Contrastive and Domain Adversarial Learning Model

To further improve the performance of the proposed model, we employed a collaborative contrastive and adversarial learning strategy that simultaneously leveraged style and domain annotations. We conducted extensive experiments with the proposed model. The experimental settings were the same as the single-level supervision models. The illustration of the architecture of the model can be observed in Figure 2, while the details can be found in the methodology in Section 3. The experimental results, as shown in Table 4, demonstrate the superior performance of our proposed model compared with other methods. The performance of the proposed model (M-Dice: 0.8999) improved by 0.37% and 0.27% compared to the single-style supervision model (M-Dice: 0.8966) and domains supervision model (M-Dice: 0.8975), respectively. Additionally, the model yielded a large improvement of 1.35% compared to the pixel-supervised-N baseline (M-Dice: 0.8879). Regarding the $CDR_{MSE}$ and G-score, our proposed model (0.0043 and 51.23) still yielded significant improvements compared to the pixel-supervised-N baseline (0.0097 and 46.97). Compared to the pixel-supervised-G baseline, our proposed model achieved a comparable performance with a slight decrease of 1.19% and 3.50% for M-Dice and G-score, respectively. The divergence in terms of the $CDR_{MSE}$ was also modest. These results prove that our proposed model can narrow the performance gap to the pixel-supervised-G baseline. In addition, compared to the common baseline-G&N (M-Dice: 0.9127), the results of our proposed model are comparable, even if they utilize high-cost pixel-level annotations from both glaucoma and normal images. Therefore, using low-cost annotated normal images but achieving a promising performance on glaucoma samples proves that the proposed model is suitable for assisting in tracking of the progression and assessment of the prognosis of glaucoma-confirmed samples.

### 5.3. Results of Varying the Sizes of Pixel-Level Annotated Normal Images

To explore the correlation between pixel-level annotated normal images and our proposed model, we increased the size of the pixel-level annotated normal images in the training dataset (100, 300, and 486). All other experimental settings were the same as the proposed model. The results, as shown in Table 5, demonstrate a notable improvement with an increase in the size of the pixel-level annotated normal images. Specifically, 300 annotated normal samples achieved an increase of approximately 0.43% compared with 100 annotated normal samples. This enhancement can be attributed to benefits from a higher baseline of 300 annotated normal images (M-Dice of pixel-supervised-N baseline: 0.9001), providing more resources to exploit and enable the model to effectively capture essential features. Therefore, the proposed model with 482 annotated images yields an M-Dice of 0.9134, even better than the pixel-baseline-G (M-Dice: 0.9107), which demonstrates the effectiveness of our proposed model.

### 5.4. The Adaptability of the Proposed Model in Other Scenarios

To explore the adaptability of our proposed model in different scenarios, we investigated the performance of our proposed model in the other two common scenarios. An illustration of the proposed model can be observed in Figure 2. All experimental settings were the same as the scenario in which there were only normal images with pixel-level annotations, except using annotated glaucoma images or using both annotated glaucoma and normal images as the source training dataset for Scenario 2 and Scenario 3, respectively.

When only the glaucoma samples had pixel-level annotations, we utilized the same training dataset as for the pixel-baseline-G model (100 glaucoma with pixel-level annotations), along with 68 unannotated glaucoma samples. Our proposed model demonstrates its adaptability to address this scenario by only using the style-supervision module. The results, as shown in Table 6, demonstrate the superior performance of our proposed model compared to the baseline pixel-supervised-N model, specifically in terms of OD and Rim. Notably, an increasing trend was also observed in the glaucoma-related metrics. These results highlight the value of the unannotated glaucoma samples, which can serve as a resource for extracting valuable features, simultaneously proving the capability of our proposed model to capture glaucoma-related features.

Moreover, in the scenario with both glaucoma and normal images with pixel-level annotations, we utilized the dataset as the same training dataset used in the pixel-baseline-G&N model (100 glaucoma and 100 normal with pixel-level annotations). The results, as shown in Table 6, demonstrate a notable improvement of 1.84% compared to the performance of the model using only pixel-level annotated normal images. This enhancement can be attributed to the benefits of additional pixel-level annotations for the glaucoma images, resulting in the model capturing more general and glaucoma-related features. Furthermore, compared to the pixel-baseline-G&N, the model achieved an increase of 0.42% for M-Dice. Therefore, even with the same annotations, our relationship-driven model demonstrated a superior capacity to disentangle the relationship between normal and glaucoma classes than the direct mixed pixel-supervised-G&N method, enabling the model to concentrate on the target glaucoma class.

### 5.5. Results for the Adapted G1020 Fundus Image Dataset

To evaluate the generalization ability of our proposed model, we conducted experiments with the challenging fundus dataset (G1020) in different situations. The experimental settings were consistent with the model for the adapted ORIGA dataset. The experimental results are shown in Table 7, demonstrating the superiority of the proposed model in both OC and rim segmentation results of 0.8912 and 0.8074, respectively. This represents significant improvements of 3.42% and 4.44% over the pixel-supervised-N baseline (OC: 0.8617; Rim: 0.7731). However, it is worth noting that our proposed model did not achieve an evident improvement in OD segmentation compared with the other baseline models. The variations in the OD, OC, and rim segmentation results may be attributed to characteristics of glaucoma, which primarily affect the OC and rim regions. Notably, regarding glaucoma-related features, our proposed model also achieved the best results of all methods. Compared to the ground truth, the CDR_MSE_ results for our proposed model were the lowest of all methods, at only 0.0068, compared to 0.0175 for the pixel-supervised-N baseline. Regarding the G-score, our proposed model continued to outperform the other methods, achieving a superior performance (45.07) to the pixel-supervised-N baseline (38.81).

### 5.6. Computational Complexity of the Models

To assess the computational complexity of the different models, a critical consideration in clinical practice, we employed the following five assessment metrics: parameters of the model, floating-point operations (FLOPs), training time required per epoch for a model, inference time needed for a single sample for an optimized model, and size of the saved optimized models. We only compared two kinds of methods, since all baseline models shared the same framework, which can be replaced by other advanced segmentation models in our proposed model. The results, as shown in Table 8, reveal that our proposed model had a slightly higher cost in terms of parameters and FLOPs over the baseline models due to the inclusion of three additional modules. As a result, the training time required for our proposed markedly surpassed that of the baseline models. Moreover, it is noteworthy that despite the high cost of the training time for our model, the inference time for both models remained consistent at 0.22 s. This consistency is attributed that the proposed model only relying on the segmentation component during the inference stage, so the size of the saved optimized model to the inference samples for both models remained the same.

## 6. Visualization

### 6.1. Visualization of the Results for Common and Challenging Samples

**Figure 3:**
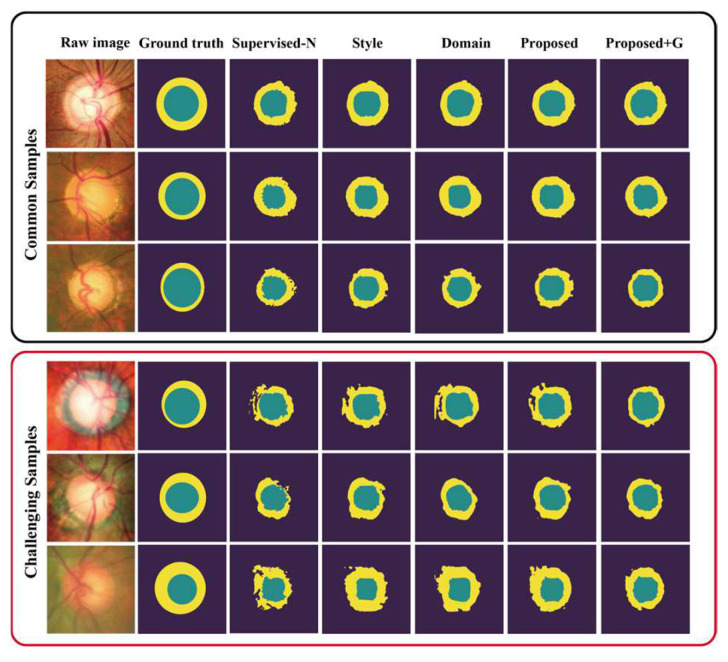
Visual comparison of segmentation results from the various models on the glaucoma samples from the ORIGA dataset. The upper three examples are common samples, while the lower three examples present challenging samples. The last method, denoted as “Proposed+G”, encompasses the proposed style and domain transfer model with annotated glaucoma and normal fundus images in Scenario 2.

**Figure 4:**
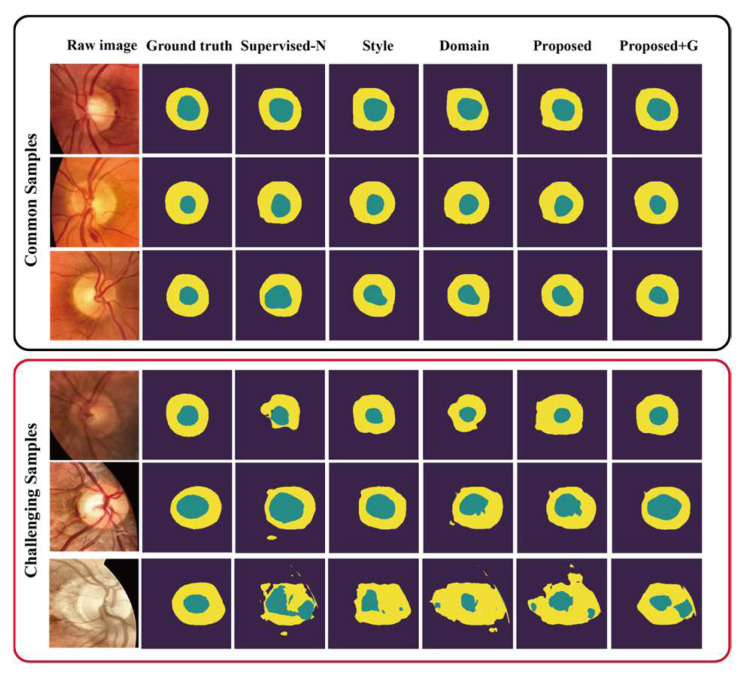
Visual comparison of the segmentation results from the various models on glaucoma samples from the G1020 dataset. The upper three examples are common samples, while the lower three examples represent challenging samples.

To facilitate the comprehensibility of the experimental results, we employed direct visualization of the segmental results obtained from different models, encompassing scenarios with and without annotations of the glaucoma samples. The visualized results for the tested glaucoma samples from ORIGA and G1020 are shown in Figure 3 and Figure 4, respectively. These results explicitly show the effectiveness of our style and domain supervision model in approximating ground truth, outperforming the pixel-supervised-N baseline. Furthermore, in both adapted datasets, our proposed model shows superior precision in the OC segmentation results, particularly in the rim segmentation results compared to the other methods. Moreover, in the situation with the annotated glaucoma samples, our proposed model showed a heightened level of accuracy in delineating the contours of OC and OD, consequently leading to an improvement in the overall accuracy of the segmentation results.

In addition, we present a visual comparison of the challenging samples to show the ability of our proposed model to address complex samples (i.e., severe glaucoma). Six challenging samples in the datasets, as depicted in the bottom rows of Figure 3 and Figure 4, effectively demonstrate that our proposed model achieves superior performance on challenging samples in comparison to the other methods. The pixel-supervised-N baseline showed a poor performance on these samples, failing to achieve an acceptable level with intact and accurate contours of the OD and OC, mainly due to significant dissimilarities between normal and severe glaucoma samples. However, notable enhancements were observed in our proposed model in both situations, showing its capacity to capture glaucoma-style and glaucoma-domain features.

### 6.2. The Distribution of the Features for Different Models

**Figure 5:**
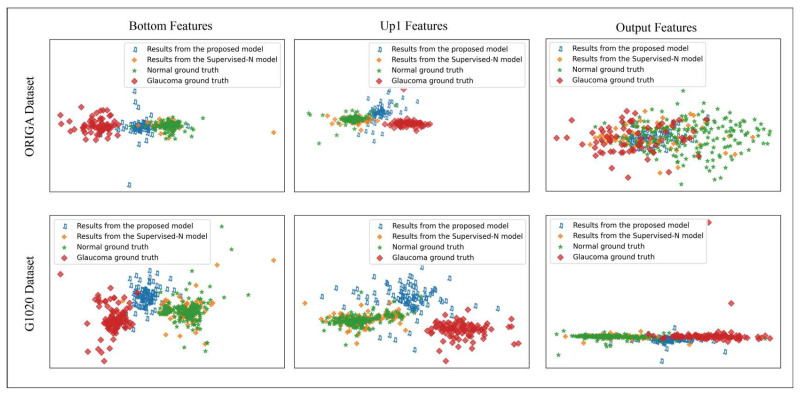
The distribution of the encoding (Bottom and Up1) and output features obtained from the various models, along with the corresponding ground truths of the normal and glaucoma classes. The three figures in the upper row are from the ORIGA dataset, while the lower three subfigures depict the results from the G1020 dataset.

To facilitate a comparative analysis of efficacy differentials across various models on a global scale, we employed a direct and insightful visualization approach to provide insight into the distribution of the prediction features. To achieve this, we delineated the distribution of the prediction features, including the features from the two encoding and the output spaces generated by the pixel-level supervision baseline models and the proposed model, using the same glaucoma samples. The obtained distributions, as shown in Figure 5, reveal a significant gap between the distribution of the prediction features from the pixel-supervised-N model and the actual glaucoma ground truth. Remarkably, the former distribution closely aligns with that of the normal ground truth. In contrast, the distribution of prediction results generated by the proposed model approaches the distribution of the glaucoma ground truth, exhibiting a significant departure from the normal distribution observed in the pixel-supervised-N baseline model. These results indicate that, in the absence of the proposed modules, only supervision by the pixel-level annotations of the normal samples proved insufficient to promote the active capture of glaucoma-related features by the pixel-supervised-N baseline model, while our proposed model exhibited a capacity to effectively capture glaucoma features in different spaces.

### 6.3. The Style Gap Between the Results and Ground Truths for the Various Models

**Figure 6:**
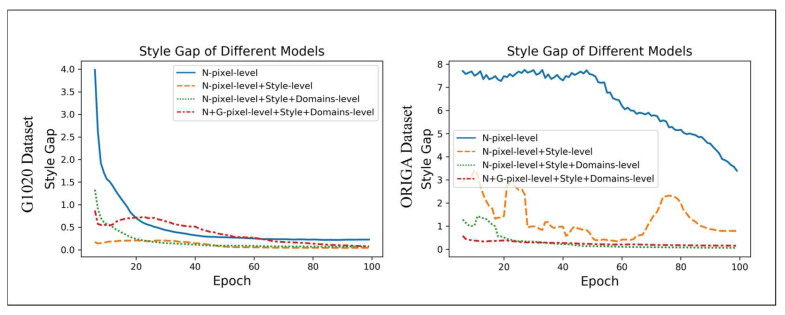
The plots of the style gaps between the results generated by the various models and corresponding ground truths during the training stage. The left figure illustrates the results from the ORIGA dataset, while the right figure corresponds to the G1020 Dataset. The N-pixel-level is the direct supervision with normal fundus images with pixel-level annotation; style-level is the proposed model with style module and style-level annotations; domain-level is the proposed model with the domain module and domain-level annotations; and G-pixel-level represents the glaucoma samples with pixel-level annotations.

To conduct a comprehensive evaluation of the efficacy in capturing glaucoma-style features across various models, we plotted the style gap between the results generated by these models and the corresponding ground truths. The results, as shown in Figure 6, demonstrate that the proposed model, integrating both style and domain modules, exhibited the minimal style gap between the prediction results and ground truths over the directly supervised model with the same pixel-level annotated normal samples. Notably, the style gap of the pixel-supervised-N baseline model also decreased progressively during training, which can be attributed to the overlap or similarity between the normal and glaucoma classes. Moreover, the results reveal that the proposed model, incorporating both style and domain modules, yielded a more consistent and stable trend than the model with only the style module, showcasing that cooperatively supervised learning from the two perspectives of style-level and domain-level annotations can improve the stability of the proposed model. Significantly, we argue that there exist some differences in the curves of the performances between the two datasets, which can be attributed to the distinct characteristics inherent in each dataset, while both datasets exhibited a similar trend in their corresponding curves.

## 7. Discussion

In our previous study [3], we leveraged OCT images to extract retinal-layer-based biomarkers by accurate segmentation of retinal layers. However, the high cost of acquiring OCT images, coupled with the necessity of comprehensively evaluating glaucoma progression highlights the need to integrate various clinical indicators beyond OCT image-based biomarkers. Thus, in this study, we obtain an additional glaucoma-related biomarkers based on accurate segmentation OD and OC from low-cost color fundus images. In clinic practice, these distinct glaucoma-related biomarkers complement each other, when used together, provide a more comprehensive tool for assisting in monitoring the development of glaucoma from two different aspects.

In this study, we aim to develop a glaucoma-specifical model suitable for assisting in tracking glaucoma progression and assessing prognosis, particularly for addressing the challenge associated with the limited availability of pixel-level annotated glaucoma images. Therefore, we propose a model to exploit wealth from sufficient and low-cost annotated normal images. We utilize annotated normal fundus images as a resource for extracting valuable features, while glaucoma fundus images as target samples to validate our proposed model from two public glaucoma detection datasets, namely ORIGA and G1020. In addition, we introduce the following three metrics to comprehensively evaluate our proposed model: Dice for segmentation accuracy, *CDR_MSE_* for glaucoma-related features, and *G-score* for both segmentation accuracy and glaucoma-related features.

We initially established the following three baselines: pixel-supervised-N baseline (training with pixel-level annotated normal fundus images); pixel-supervised-G baseline (training with pixel-level annotated glaucoma fundus images); and mixed pixel-supervised-G&N baseline (training with glaucoma and normal images with pixel-level annotations). Subsequently, we established and systematically validated our proposed model with the adapted datasets. Through a comprehensive analysis of the experimental results, our proposed model can be adapted to various situations. Notably, in the scenario of only normal images with pixel-level annotations, our proposed model achieved a superior performance over the pixel-supervised-N baseline while approaching that of the pixel-supervised-G baseline. Specifically, the prediction results for the OC and optic rim significantly outperformed the pixel-supervised-N baseline in terms of all three metrics, while the OD performance remained almost the same. The observed difference in the results for the OD and OC matches the inherent characteristics of glaucoma pathology. Furthermore, compared with the pixel-mixed-G&N baseline, our proposed model consistently exhibited comparable performance, affirming the effectiveness of our proposed model. Additionally, we found a significant improvement with the increase in size of the pixel-level annotated normal images, attributed to a greater number of available annotations, enabling the model to effectively capture essential features. When we only had glaucoma samples with pixel-level annotations and some glaucoma samples without pixel-level annotations, our proposed model yielded a better performance than the pixel-supervised-N model baseline by capturing glaucoma-related features from the unannotated glaucoma samples.

In the case involving limited pixel-level annotated glaucoma and normal images, our proposed model exhibited a capacity to further improve in performance, yielding better results than the pixel-supervised-G&N baseline. These results highlight its potential in addressing the challenge of the insufficient availability of annotated glaucoma images. It also proves that detection or screening models, trained with mixed data with a direct supervised strategy, is not the optimal choice for glaucoma samples and assisting in monitoring the progression and assessing the prognosis of glaucoma because of the high cost but marginal performance gains. Therefore, our relationship-driven model effectively enhances performance by disentangling the relationship between glaucoma and normal classes.

Moreover, we present in-depth insight into the role of style and domain supervision in our proposed model. The model with only style or domain supervision modules achieved superior performance compared to the pixel-supervised-N baseline. Notably, the domain supervision model can achieve better results than the style supervision model. However, implementation of the style supervision by Gram transformers is easier than the domain supervision learning. Certainly, they each rely on distinct information to capture glaucoma-style and glaucoma-domain features, respectively. We argue that low-cost supervision at a single level is insufficient for pixel-level prediction tasks. Therefore, integrating advantages from the style and domain annotations using a cooperative supervision strategy can exploit simultaneously valuable features from style and domain information, surpassing the capabilities of all single-supervision models. While raw glaucoma images have pixel-level annotations, our proposed model demonstrates the capability with or without those annotated glaucoma samples.

The visualization results of the test samples in both situations directly demonstrate that our proposed model can capture target glaucoma-style and glaucoma-domain features, approaching the target glaucoma class more than the pixel-supervised-N baseline for both adapted datasets. In addition, our proposed model also showed a superior performance on challenging samples. In summary, our proposed model can capture general features by leveraging style- and domain-based similarities from low-cost pixel-level annotated normal samples. Simultaneously, it exploits dissimilarities in style and domain to capture glaucoma-related features and mitigate the impact of noised features.

Therefore, our proposed model can yield greater accuracy in delineating the OD and OC boundaries in glaucoma-confirmed samples compared to existing models. By utilizing these delineated boundaries, we can precisely calculate OC and OD diameters, as well as their ratio, and provide morphological visualizations. As these markers are a critical indicator for assessing the severity level of glaucoma, our proposed model serves as a valuable tool for assisting in assessing the development of glaucoma compared with other existing models.

Nevertheless, our proposed model still has certain limitations. Firstly, our proposed model is not intended for direct application in the tracking of glaucoma progression; rather, it aims to assist in the assessment of glaucoma progression by providing precise segmentations of OD and OC in glaucoma-confirmed samples, as accurate assessment of the progression of glaucoma depends on various clinical evidence, including visual field assessment and intraocular pressure, rather than solely relying on CDR values. Moreover, our proposed model is premised on patients with confirmed glaucoma, as the model is designed to specifically assist in monitoring progression and assessment of the prognosis of glaucoma with fundus images, making it unsuitable for scenarios of glaucoma screening and detection. Furthermore, our proposed model exhibits a performance gap compared to the pixel-supervised-G baseline when without pixel-level annotated glaucoma samples, deteriorating its practicality in clinic practice. Moreover, it shows modest performance on challenging glaucoma images, restricting its applicability in diverse real-world scenarios, and it may exhibit a limited contribution to algorithm development, as both of the main employed techniques are already commonly used for various tasks.

In the near future, we aim to mitigate the modest performance observed on the challenging samples, with a focus on approximating the performance of the pixel-supervised-G baseline when without annotated glaucoma samples. Furthermore, we plan to exploit normal samples from different vendors, adapting various scenarios. Additionally, we plan to collect a time-serial glaucoma monitoring progression dataset by continuously tracking fundus images of the patients during their therapy, thus enabling a comprehensive evaluation of the performance of glaucoma. Moreover, conducting an experiment to compare the time required for annotating two sample classes (glaucoma and normal) and to quantify the cost-effectiveness brought by normal samples represents a valuable avenue for future research. In our future work, we aim to design a novel framework that leverages the features associated with glaucoma progression, which may improve performance on assisting in assessing glaucoma progression.

## 8. Conclusions

In this paper, we address the challenge of the high cost of developing a glaucoma-specialized model to assist in monitoring glaucoma progression and prognosis assessment by exploiting low-cost annotated normal fundus images with an annotation-efficient approach. We propose a cost-efficient model to capture features from style and domain annotations between two classes. Our proposed model is suitable for various scenarios including with or without annotated glaucoma images. The experimental results on two adapted datasets demonstrate that our proposed model achieves a significantly superior performance in both segmentation- and glaucoma-related metrics compared to the baseline model. In addition, the visualization of the results provides direct evidence illustrating the advantages of our proposed model. The proposed model can yield performance improvements and cost reductions simultaneously. We hope that our proposed model offers valuable contributions to preventing vision loss caused by glaucoma.

## Figures and Tables

**Table 1:** Detailed architecture of the proposed model.

Feature Size	Pixel-level Path		Class-level Part			
3 × 256 × 256	Input		Domain Supervision Path			
64 × 128 × 128	Down1	Double-Conv-Maxpool	256 × 256 × 256	Input		3 × 256 × 256
128 × 64 × 64	Down2	Double-Conv-Maxpool	3 × 128 × 128	D-Conv1	Conv(4 × 4)-Leak-ReLU	3 × 128 × 128
256 × 32 × 32	Down3	Double-Conv-Maxpool	6 × 64 × 64	D-Conv2	Conv(4 × 4)-Leak-ReLU	6 × 64 × 64
512 × 16 × 16	Down4	Double-Conv-Maxpool	12 × 32 × 32	D-Conv3	Conv(4 × 4)-Leak-ReLU	12 × 32 × 32
512 × 16 × 16	Bottom	Double-Conv	24 × 32 × 32	D-Conv4	Conv(3 × 3)-Leak-ReLU	24 × 32 × 32
256 × 32 × 32	Up1	Upsample-Double-Conv	1 × 32 × 32	Output	Conv(3×3)	1 × 32 × 32
128 × 64 × 64	Up2	Upsample-Double-Conv	Style Supervision Path			
64 × 128 × 128	Up3	Upsample-Double-Conv	2 × (3 × 256 × 256)	Input		
32 × 256 × 256	Up4	Upsample-Double-Conv	2 × (3 × 3)	Transform	Gram matrix	
3 × 256 × 256	Output	Conv(3 × 3)	1 × 1	Output	MSE	

Double-Conv: Conv2D (3 × 3) → BatchNorm → ReLU → Conv2D (3 × 3) → BatchNorm → ReLU.

**Table 2:** Summary of the two adapted datasets.

Dataset		ORIGA	G1020
Category		Fundus image	Fundus image
	Normal	482	724
	Glaucoma	168	296
Eye			
	Right	314	490
	Left	336	530
Image Size		2000 × 3000 × 3	2400 × 3000 × 3
Annotation	OD & OC	650	790

OD & OC: the samples have pixel-level annotations for the OD and OC regions. The sizes of the images in the two datasets are not fixed but only vary slightly; therefore, we provide an approximate value.

**Table 3:** Results of the pixel-level supervision baseline models.

Baseline Models	Dice				$CDR_{MSE}$	G-Score
	OD	OC	Rim	Mean		
Scenario 1: Pixel-Supervised-N	0.9368	0.9158	0.8111	0.8879	0.0097	46.97
Scenario 2: Pixel-Supervised-G	0.9516	0.9308	0.8497	0.9107	0.0028	53.09
Scenario 3: Pixel-Supervised-N&G	0.9518	0.9329	0.8534	0.9127	0.0022	54.10

Pixel-supervised-N: the model is supervised by all pixel-level annotated normal fundus images. Pixel-supervised-G: the model is supervised by all pixel-level annotated glaucoma fundus images. Pixel-supervised-N&G: the model is supervised by mixed pixel-level annotated glaucoma and normal fundus image.

**Table 4:** Results of different levels of the supervision models.

Transfer Learning			Dice				$CDR_{MSE}$	G-Score
Contrastive	Adversarial		OD	OC	Rim	Mean		
Style	Output	Encoding						
✔			0.9394	0.9223	0.8281	0.8966	0.0090	49.85
	✔		0.9416	0.9176	0.8244	0.8945	0.0081	50.83
		✔	0.9429	0.9107	0.8233	0.8923	0.0070	49.67
	✔	✔	0.9413	0.9228	0.8284	0.8975	0.0080	50.86
✔	✔	✔	0.9341	0.9397	0.8315	0.8999	0.0043	51.23

Output or encoding is domain-level supervision in encoding or output spaces by adversarial learning, respectively. Style is style-level supervision by contrastive learning. A checkmark indicates that the corresponding learning is adopted.

**Table 5:** Results of differently sized pixel-level annotated normal images.

Size (A–N)	Model	Dice				$CDR_{MSE}$	G-Score
		OD	OC	Rim	Mean		
100	Pixel-Supervised-N	0.9368	0.9158	0.8111	0.8879	0.0097	46.97
	Proposed Model	0.9341	0.9397	0.8315	0.8999	0.0043	51.24
300	Pixel-Supervised-N	0.9471	0.9219	0.8313	0.9001	0.0056	49.42
	Proposed Model	0.9487	0.9234	0.8393	0.9038	0.0050	51.91
482	Pixel-Supervised-N	0.9550	0.9219	0.8469	0.9080	0.0053	52.35
	Proposed Model	0.9527	0.9331	0.8546	0.9134	0.0029	53.32

Note that A–N means pixel-level annotated normal fundus images in Scenario 1, wherein only normal fundus images have pixel-level annotations.

**Table 6:** Results of the proposed model with annotated glaucoma and normal images.

Scenario	Model	Dice				$CDR_{MSE}$	G-Score
		OD	OC	Rim	Mean		
Scenario 2	Pixel-Supervise-G	0.9516	0.9308	0.8497	0.9107	0.0028	53.09
	The proposed model	0.9539	0.9397	0.8499	0.9145	0.0021	54.50
Scenario 3	Pixel-Supervised-G&N	0.9518	0.9329	0.8534	0.9127	0.0022	54.10
	The proposed model	0.9541	0.9374	0.8580	0.9165	0.0022	54.25

Scenario 2: only glaucoma fundus images have pixel-level annotations. Pixel-supervised-G&N: a directly supervised strategy was employed using all pixel-level annotated glaucoma and normal samples. Scenario 3: both normal and glaucoma fundus images have pixel-level annotations. Pixel-supervised-G: a directly supervised strategy was employed using all pixel-level annotated glaucoma samples.

**Table 7:** Results of the proposed model on the G1020 dataset.

Scenario	Model	Dice				$CDR_{MSE}$	G-Score
		OD	OC	Rim	Mean		
Scenario 1	Pixel-Supervised-N	0.9559	0.8617	0.7731	0.8636	0.0175	38.81
	The proposed model	0.9583	0.8912	0.8074	0.8860	0.0068	45.07
Scenario 2	Pixel-Supervised-G	0.9544	0.8889	0.7974	0.8802	0.0094	43.61
	The proposed model	0.9547	0.8866	0.8048	0.8820	0.0085	43.30
Scenario 3	Pixel-Supervised-G&N	0.9651	0.8993	0.8081	0.8960	0.0061	45.45
	The proposed model	0.9656	0.9071	0.8315	0.9014	0.0047	46.19

Scenario 1: only normal fundus images have pixel-level annotations. Scenario 2: only glaucoma fundus images have pixel-level annotations. Scenario 3: both normal and glaucoma fundus images have pixel-level annotations. Baseline models: Pixel-supervised-N and pixel-supervised-G were trained with pixel-level annotated normal and glaucoma fundus images, respectively. While the pixel-supervised-G&N model was directly supervised by pixel-level annotated normal and glaucoma fundus images. The proposed model: the model with style- and domain-level supervision modules.

**Table 8:** The computational complexity of the proposed model.

Method	Parameters (M)	FLOPs (G)	Train Time (Min)	Infer Time (s)	Saved Model (M)
Baseline models	31.04	40.93	9.48	0.22	130
Our proposed model	31.08	41.76	34.10	0.22	130

## Data Availability

Data underlying the results presented in this paper are available in Refs. [51,52].

## Supplementary Materials

The following supporting information can be downloaded at: https://www.mdpi.com/article/10.3390/s24227255/s1, Figure S1: (left) The color fundus image was captured of normal people; (right) the color fundus image was of a patient with glaucoma. A, B, and C are pixels in the image; Figure S2: The proposed model adapts Scenario 1 (only normal images have pixel-level annotations); Figure S3: The proposed model adapts Scenario 2 (only glaucoma images have pixel-level annotations); Figure S4: The proposed model adapts Scenario 3 (both normal and glaucoma images have pixel-level annotations); Figure S5: An illustration of our preprocessing steps for the color fundus images, including extracting ROIs based on the optic disc labels and employing an adaptive histogram equalization algorithm to enhance contrast ratio; Table S1: Results of different transfer models in the G1020 Dataset.
