# Supplementary material for: Development of a Cost-Efficient and Glaucoma-Specialized OD/OC Segmentation Model for Varying Clinical Scenarios
Source: Sensors (Basel). 2024 Nov 13;24(22):7255. doi: 10.3390/s24227255 (PMC11597940; doi:10.3390/s24227255)
Supplement: Supplementary file 1 [file sensors-24-07255-s001.zip › sensors-3263580-supplementary.pdf]

Development of a cost-efficient and glaucoma-specialized OD/OC segmentation model for varying clinical scenarios : supplemental document

To make it easier to understand the various categories of labels, we present the following example. In the left illustration of Fig. S1, the pixel-level annotation for pixel at position A is classified as the background, while the pixel-level annotation for pixel at position B is categorized as optic cup. The style-level annotation of this image belongs to the normal style, and the label of the domain is categorized as the normal domain. Conversely, in the right illustration of Fig. S1, the pixel-level annotation of pixel C is also classified as optic cup; however, the style-level annotation of this image is designated as glaucoma style, with the domain label corresponding to the glaucoma domain.

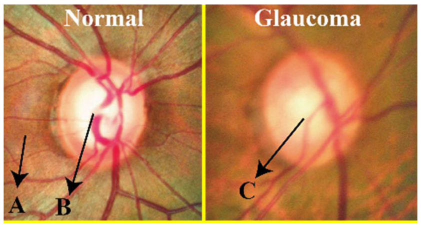

Figure S1. The left color fundus image is captured from normal people, while right color fundus image is captured from glaucoma patient. A, B, and C is a pixel in the image.

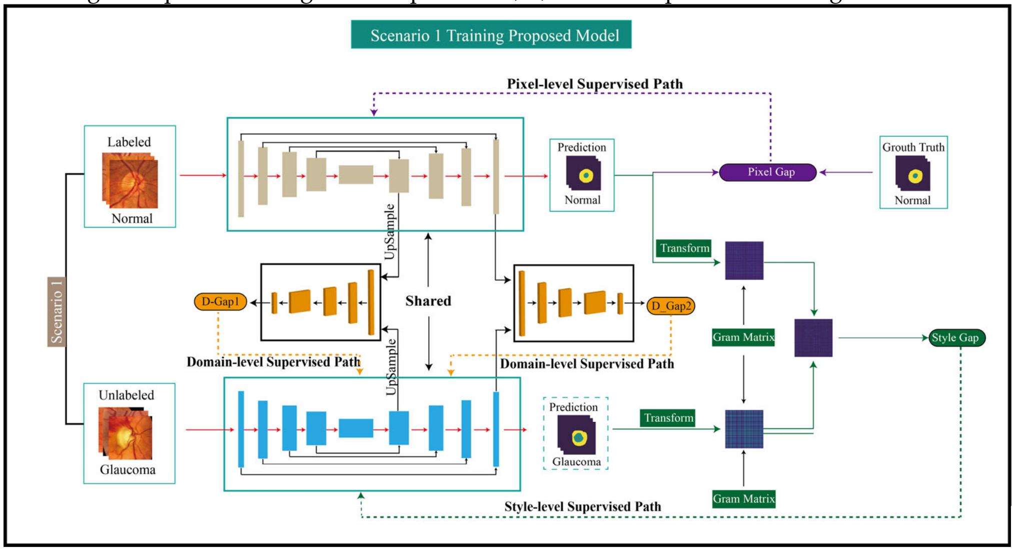

Figure S2. The proposed model adapts scenario 1: only normal images have pixel-level annotations.

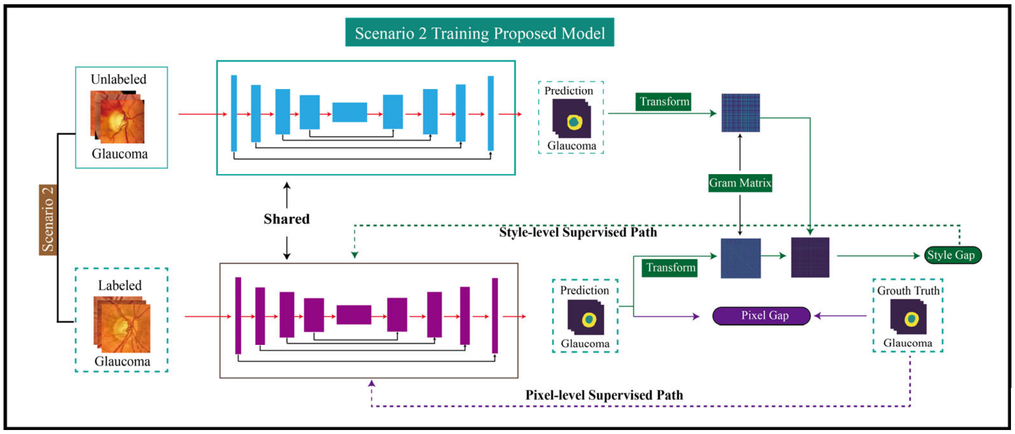

Figure S3. The proposed model adapts scenario 2: only glaucoma images have pixel-level annotations.

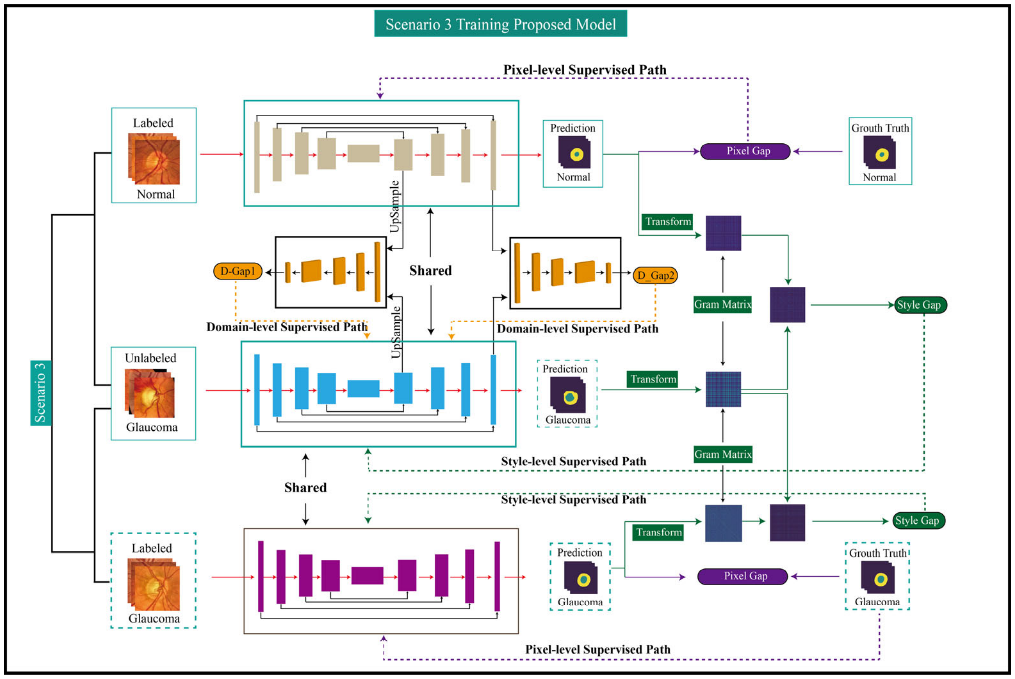

Figure S4. The proposed model adapts scenario 3: both normal and glaucoma images have pixel-level annotations.

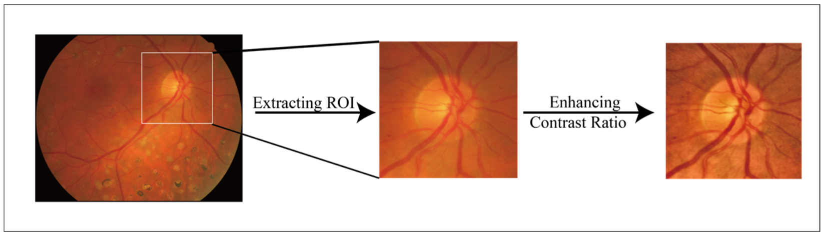

Figure S5. The illustration of our preprocess steps for color fundus images, including extracting ROI region based on optic disc labels, and employing adaptive histogram equalization algorithm to enhance contrast ratio.

Table S1. Results of Different Transfer Models in G1020 Dataset.

Module			Dice				CDR_{MSE}	G-score
Style	Output	Encoding	OD	OC	Rim	Mean		
✔			0.9584	0.8880	0.8009	0.8824	0.0090	44.77
	✔		0.9518	0.8811	0.7982	0.8771	0.0087	44.42
	✔	✔	0.9566	0.8852	0.7992	0.8803	0.0084	44.55
✔	✔	✔	0.9583	0.8912	0.8074	0.8860	0.0068	45.07
